# Precision in Prevention and Health Surveillance: How Artificial Intelligence May Improve the Time of Identification of Health Concerns through Social Media Content Analysis.

**DOI:** 10.1055/s-0044-1800736

**Published:** 2025-04-08

**Authors:** Pascal Staccini, Annie Y.S. Lau, Findings from the Yearbook 2024 Section on Consumer Health Informatics

**Affiliations:** 1 URE RETINES, Faculté de Médecine, Université Côte d'Azur, Nice, France; 2 Center for Health Informatics, Australian Institute of Health Innovation, Macquarie University, Australia

**Keywords:** Consumer Health Informatics, Social media, Health Surveillance, Mental Health, Artificial Intelligence, Machine Learning, Natural Language Processing, Public Health Informatics

## Abstract

**Objective** : To explore how artificial intelligence (AI) methodologies, particularly through the analysis of social media content, can enhance “precision in prevention and health surveillance” (2024 Yearbook topic). The focus is on leveraging advanced data analytics to improve the timeliness and accuracy of identifying emerging health concerns, thus enabling more proactive and effective health interventions.

**Methods** : A comprehensive literature search strategy was conducted on PubMed, focusing on papers published in 2023 related to consumer health informatics, precision prevention, and the intersection with social media. The search aimed to identify studies that utilized AI and machine learning techniques to analyse social media data for health surveillance purposes. Bibliometric analyses were applied to the retrieved articles, and tools such as “Bibliometrix” were used to assess keyword frequencies, co-occurrence networks, and thematic maps. The studies were then independently reviewed and screened for relevance, with a final selection of 10 articles made based on their alignment with the 2024 Yearbook topic and their methodological innovation.

**Results** : The analysis of 89 articles revealed several key themes and findings. Social media data offers a rich source of real-time insights into public health trends, and encompasses diverse demographic groups. AI methodologies, including machine learning, natural language processing (NLP), and deep learning, play a crucial role in extracting and analysing health-related information from social media content. The integration of AI in health surveillance can provide early warnings of potential health crises, as demonstrated by studies on topics such as suicide prevention, mental health, and the impact of social media use on e-cigarette consumption among youth. Ethical and privacy considerations are paramount, necessitating robust data anonymization and transparent data handling practices. Advanced AI techniques, such as transformer-based topic modelling and federated learning, enhance the precision and security of health surveillance systems. The document highlights several case studies that demonstrate the practical applications of AI in health surveillance, such as monitoring public discussions about delta-8 THC and assessing suicide-related tweets and their association with help-seeking behaviour in the US.

**Conclusion** : Integrating AI and social media content analysis in precision prevention and health surveillance has significant potential to improve public health outcomes. By leveraging real-time, comprehensive data from social media platforms, AI can enhance the timeliness and accuracy of identifying health concerns. Addressing ethical and privacy challenges is crucial to ensure responsible and effective implementation. The continuous advancement of AI technologies will play a critical role in safeguarding public health and responding to emerging health threats.

## 1. Introduction

Precision in prevention and health surveillance represents a transformative approach in public health, leveraging advanced data analytics and artificial intelligence (AI) to enhance the timeliness and accuracy of identifying health concerns. The use of AI methodologies in analyzing social media content has emerged as a crucial component in this field, providing real-time insights into public health trends and enabling more proactive health interventions.

One of the significant advantages of integrating AI into health surveillance is its ability to process vast amounts of unstructured data from social media platforms. Social media generates a continuous stream of user-generated content that reflects public sentiment, behaviors, and experiences. This data can be invaluable for detecting emerging health threats and monitoring public responses to health interventions. For instance, a study utilized transformer-based topic modeling to examine discussions about delta-8 tetrahydrocannabinol (THC) on Reddit, identifying health-related symptoms and safety concerns associated with this substance [ 1 ]. This approach demonstrates how AI can uncover emerging health issues by analyzing social media discourse.

Moreover, social media platforms provide a rich source of data that encompasses diverse demographic groups. This inclusivity is essential for tailoring public health interventions to specific populations. The analysis of social media data can reveal patterns and trends that are not readily apparent through traditional data sources. For example, research on social distancing behaviors during the COVID-19 lockdown in South Africa highlighted the importance of understanding public compliance and attitudes towards preventive measures [ 2 ]. Such insights are critical for developing targeted health communication strategies.

Machine learning algorithms, particularly those using natural language processing (NLP), play a pivotal role in extracting relevant information from social media content. These algorithms can identify and classify health-related topics, sentiments, and trends, providing early warnings of potential health crises. An example is a study that developed a machine learning model to estimate firearm homicides in near real-time, illustrating the predictive capabilities of AI in public health surveillance [ 3 ]. Similarly, NLP has been used to analyze the association between social media use and mental health issues, such as anxiety and depression, among youth, highlighting the impact of social media on public health [ 4 ].

The integration of deep learning techniques further enhances the precision of health surveillance systems. Deep learning models can analyze complex patterns in data, improving the detection of health-related signals from social media. For instance, a study on the prevalence of PTSD symptoms among healthcare workers during the COVID-19 pandemic used deep learning to identify psychological risk factors and protective factors, demonstrating the utility of AI in mental health surveillance [ 5 ].

Ethical and privacy considerations are paramount when utilizing social media data for health surveillance. The collection and analysis of personal data from social media platforms raise significant privacy concerns. Ensuring data anonymization and maintaining transparency in data handling practices are crucial for protecting user privacy and maintaining public trust. Ethical guidelines and robust regulatory frameworks are necessary to address these challenges and ensure responsible use of AI in health surveillance [ 1 ].

Several case studies highlight the practical applications of AI in health surveillance. For example, research on the impact of social media use on e-cigarette use among American youth revealed significant racial differences in the mediation effects of mental health problems, suggesting the need for tailored interventions based on demographic factors [ 6 ]. Another study demonstrated the use of AI-driven analysis to monitor public discussions about the herpes zoster vaccine in Saudi Arabia, providing insights into public awareness and acceptance of vaccination [ 7 ].

Advanced AI techniques such as federated learning and explainable AI hold promise for further enhancing health surveillance systems. Federated learning allows AI models to be trained on decentralized data, improving privacy and security while maintaining analytical capabilities. Explainable AI provides transparent and interpretable results, enhancing trust and usability in health surveillance applications. A study on injury prevention strategies in community-level rugby illustrated the benefits of using AI to analyze and interpret complex data from multiple sources, informing effective intervention strategies [ 8 , 9 ].

Integrating social media data with other data sources, such as electronic health records (EHRs), wearable devices, and environmental data, can provide a comprehensive view of public health. This holistic approach enables more accurate and timely detection of health trends and outcomes. For instance, a study on adherence to hemodialysis therapy among patients with end-stage renal disease in Saudi Arabia highlighted the importance of integrating social support data with clinical data to improve patient outcomes [ 10 ].

The integration of AI and social media content analysis in precision prevention and health surveillance offers significant potential for improving public health outcomes. By leveraging real-time, comprehensive data from social media platforms, AI can enhance the timeliness and accuracy of identifying health concerns. Addressing ethical and privacy challenges is crucial to ensuring responsible and effective implementation. As AI technologies continue to evolve, their application in health surveillance will play a critical role in safeguarding public health and responding to emerging health threats. How can AI methodologies be further refined to improve the accuracy and reliability of health surveillance systems?

## 2. Methodology

### 2.1. Search Strategy

We used PubMed to conduct our search, capturing papers on consumer-using technologies and prevention concern published in the year 2023. Regarding our section CHI, the intersection between social media and precision in prevention could be structured as followed:

- identifying at-risk individuals: social media platforms can analyze user data, including posts, comments, and interactions, to identify signals indicative of mental health concerns such as depression or anxiety. Machine learning algorithms can be trained to recognize patterns in language, sentiment, and online behavior associated with mental health issues;
- targeted interventions: once at-risk individuals are identified, precision prevention strategies can be implemented. Social media platforms can deliver targeted content, resources, and support to these individuals. This could include awareness campaigns, access to online counseling services, or community support groups;
- personalized engagement: social media can facilitate personalized engagement by tailoring the content and timing of interventions based on individual preferences and behaviors. For example, sending supportive messages during times of increased online activity or providing resources in the individual's preferred language;
- monitoring and feedback loop: continuous monitoring of user interactions on social media can provide valuable data for assessing the effectiveness of precision prevention interventions. Platforms can analyze engagement metrics, user feedback, and changes in online behavior to refine and improve the targeted strategies over time.

The original search query built with the concepts listed above is detailed below (5 parts):

The complete query retrieved only one article. By removing the fourth part of the query, only two articles were found. Then by removing the third part, 92 articles were found, but only 89 were in full text. The other combinations of the remaining parts were not relevant regarding the topic. Therefore, we kept the following query as the final query (89 full text papers):

### 2.2. Bibliometric Analyses

To understand the state of the literature, we applied various bibliometrics tools onto the original set of articles returned from the search query. The “Bibliometrix” package from R [ 11 ] was used on the citation set of retrieved articles. We reported frequency of keywords. We illustrated the analysis of abstracts (measure of word frequency) by a word cloud drawing feature. We analysed keywords to uncover links between concepts through co-occurrences network. We also plotted a thematic map to analyse these clusters according to the quadrant in which they are placed [ 12 ].

- Themes in the *upper-right* quadrant are both well-developed and important for the structuring of a research field. They are known as the motor-themes of the specialty given that they present strong centrality and high density.
- Themes in the *upper-left* quadrant have well-developed internal ties but less-developed external ties and so are of only marginal importance to the field. These themes are very specialized and peripheral in character.
- Themes in the *lower-left* quadrant are both weakly-developed and marginal. The themes of this quadrant have low density and low centrality, mainly representing either emerging or disappearing themes.
- Themes in the *lower-right* quadrant are important for a research field but are not as well-developed. Each theme is represented as a sphere, its volume being proportional to the number of documents associated with the theme.

## 3. Results

### 3.1. State of the Literature

**Figure 1.**
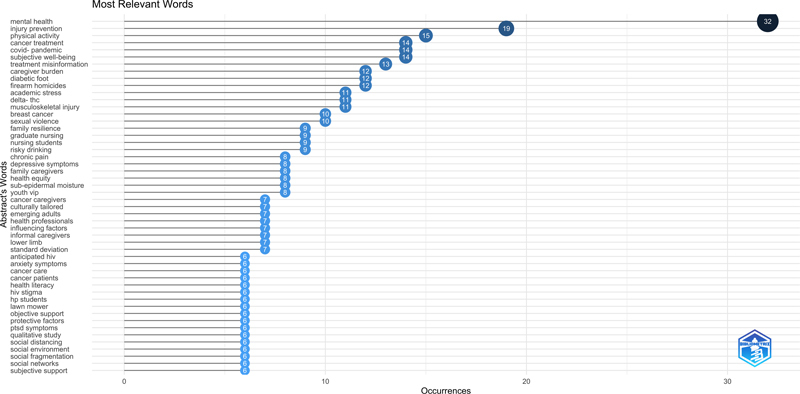
Occurrences of bigrams in abstracts of the first selection of 89 papers.

A descriptive analysis of 89 articles was conducted, reporting the frequency of keywords, and the frequency of words in titles and abstracts. 485 distinct bigrams in titles and 5,316 bigrams in abstracts were used. Figure 1 lists the 50 most frequently cited bigrams in abstracts ranked from most frequently to least frequently reported. The top ten is composed of: mental health (32 times), injury prevention (19 times), perceived social (19 times), physical activity (15 times), cancer treatment (14 times), covid-pandemic (14 times), subjective well-being (14 times), treatment misinformation (13 times), caregiver burden (12 times), diabetic foot (12 times) and firearm homicides (12 times).

**Figure 2.**
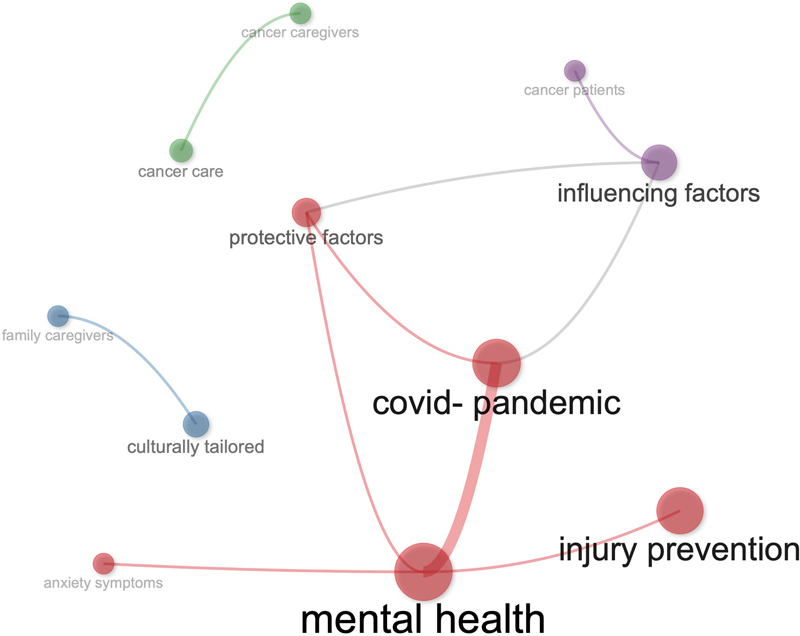
Classification of values identified in the studies.

Regarding the conceptual structure of the set of 89 articles, Figure 2 shows the co-occurrences of bigrams. Four blocks of co-occurrences have been identified. The biggest one is centred on “mental health”.One is centered on “influencing factors”. The remaining two identify “culturally tailored” and “cancer care”.

**Figure 3.**
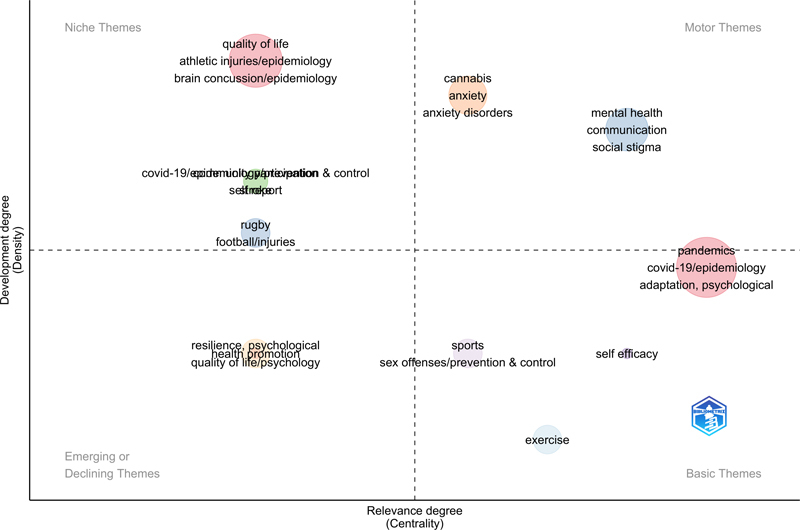
Thematic map of clusters of keywords (original set of 89 papers)

Regarding thematic maps of keywords ( Figure 3 ), clusters according to centrality (relevance degree) and density (development degree) are reported in each quadrant. The most common motor themes found across papers are related to “cannabis and anxiety disorders” and “mental health, social stigma”. Niche themes revealed by this analysis are represented by “quality of life, athletic injuries and brain concussion”. The cluster “pandemics, covid-19” is classified as basic theme.

The analysis of these first metrics allows us to see that the term at the intersection of “consumer health information” and “precision in prevention” and “health surveillance” is “mental health”.

### 3.2. Best Paper Selection

To identify the 10 candidate papers, co-editors independently assessed the 89 retrieved papers using the Rayyan web tool [ 13 ], followed by a discussion. Elements that were considered in the screening decision include 1) the level of relevance regarding the 2024 Yearbook topic “precision in prevention and health surveillance”; 2) whether the study was focused only on patients and consumers; 3) the nature of the issues addressed; and 4) level of innovative approach and methodological design. The 10 articles were then presented to a panel of international experts for full paper review and scoring according to the IMIA Yearbook best paper selection process. The final selection of best papers was completed after discussions at the annual IMIA Yearbook board meeting. For each of the 10 articles (sorted by ascending PMID), we list below the main characteristics used for the analysis and the occurrences of the keywords ( Table 1 ).

- Article 1 (PMID- 35549779) [ 2 ]: *Theme:* Social distancing behaviour during COVID-19 lockdown in South Africa. *Purpose:* This study assesses adherence to social distancing and its associated factors during the COVID-19 lockdown in South Africa. *Relevance:* Identifying specific factors that influence adherence allows for tailored public health interventions to improve compliance and reduce transmission rates. *Innovative Methods:* Utilizes logistic regression models to analyse the association between social distancing and various explanatory variables.
- Article 2 (PMID- 36239594) [ 14 ]: *Theme:* Analysis of suicide-related tweets and their association with help-seeking behaviour in the US. *Purpose:* The study assesses how different types of suicide-related content on Twitter are linked to calls to the National Suicide Prevention Lifeline and suicide rates. *Relevance:* By monitoring social media content, public health officials can identify and respond to emerging trends in suicidal behaviour more accurately and timely. *Methodology:* Uses machine learning to categorize tweets and seasonal autoregressive integrated moving average (SARIMA) analyses to assess temporal associations.
- Article 3 (PMID- 36930150) [ 3 ]: *Theme:* Near real-time estimation of firearm homicides in the US using multiple data sources. *Purpose:* The article develops a model to estimate weekly and annual firearm homicides using diverse data sources like search trends and emergency department visits. *Relevance:* Timely and accurate estimation of firearm homicides enables quicker public health responses and policy adjustments to address violence. *Methodology:* Employs machine learning and ensemble modelling to achieve high accuracy in homicide predictions.
- Article 4 (PMID- 37652109) [ 4 ]: *Theme:* Impact of social fragmentation on deaths due to alcohol, drug use, and suicide in Canada. *Purpose:* This research explores how social fragmentation correlates with mortality from alcohol use, drug use, and suicide. *Relevance:* Understanding the effects of social fragmentation helps in designing targeted interventions to improve social cohesion and reduce mortality from these causes. *Methodology:* Utilizes Cox proportional hazard regression to analyse longitudinal data.
- Article 5 (PMID- 37932203) [ 8 ]: *Theme:* Analysis of tackle characteristics and risk factors for high tackles in amateur rugby. *Purpose:* The study describes the characteristics of tackles and identifies factors associated with illegal high tackles in amateur rugby. *Relevance:* Identifying risk factors for high tackles informs preventive strategies to reduce concussion and injury rates in sports. *Methodology:* Uses video surveillance and descriptive statistics to code and analyse tackle events.
- Article 6 (PMID- 38030441) [ 9 ]: *Theme:* Evaluation of player and referee behaviour during a lowered tackle height law trial in rugby. *Purpose:* This article evaluates how a new rule regarding tackle height affects player and referee behaviours in amateur rugby. *Relevance:* Monitoring behaviour changes due to rule modifications provides insights into effective injury prevention strategies. *Methodology:* Combines video analysis with professional referee assessments to validate behavioral changes.
- Article 7 (PMID- 38065482) [ 5 ]: *Theme:* PTSD symptoms among healthcare workers during the COVID-19 surge in China. *Purpose:* The study investigates the prevalence and factors influencing PTSD symptoms in healthcare workers exposed to high workloads and infection risks. *Relevance* : Identifying stressors and protective factors among healthcare workers helps tailor mental health interventions to support them during pandemics. *Methodology:* Uses convenience sampling and logistic regression to determine the influencing factors of PTSD symptoms.
- Article 8 (PMID- 38127427) [ 1 ]: *Theme:* Discussions of delta-8 THC on social media and emerging health concerns. *Purpose:* The article analyses discussions about delta-8 THC on Reddit to identify trends and health concerns related to this cannabinoid. *Relevance:* Early detection of health issues through social media monitoring helps in timely public health interventions. *Methodology:* Applies transformer-based topic modelling for analysing unstructured social media data.
- Article 9 (PMID- 37561096) [ 15 ]: *Theme:* Depression and anxiety symptoms among hospital-based healthcare workers during COVID-19 in China. *Purpose:* This research examines the prevalence and factors influencing depressive and anxiety symptoms in healthcare workers during a COVID-19 surge. *Relevance:* Understanding mental health challenges in healthcare workers enables the development of precise interventions to support their well-being. *Methodology:* Uses validated questionnaires (PHQ-9 and GAD-7) and ordinal logistic regression for analysis.
- Article 10 (PMID- 38169240) [ 6 ]: *Theme:* Link between social media use and e-cigarette use among American youth. *Purpose:* The study explores how social media use is associated with e-cigarette use among youth, considering mental health issues as mediators. *Relevance:* Identifying the pathways linking social media use to risky behaviours helps create targeted prevention strategies. *Methodology:* Employs generalized structural equation models to examine mediation pathways.

**Table 1.** List of the top ten keywords of the 10 selected articles.

Words	Occurrences
covid-19/epidemiology	2
pandemics	2
anxiety	1
anxiety disorders	1
anxiety/epidemiology	1
athletic injuries/prevention & control/epidemiology	1
cannabidiol	1
cannabis	1
communicable disease control	1
covid-19/epidemiology/prevention & control	1

All these 10 articles contribute to the theme of “precision in prevention and health surveillance” by:

- Identifying specific risk factors: by pinpointing specific behaviours, demographics, and environmental factors that influence health outcomes, these studies provide actionable insights for targeted interventions. Articles 3, 4, 5 and 6 focus on identifying risk factors (firearm homicides, social fragmentation, sports injuries) and implementing preventive measures. Articles 7, 8 and 9 emphasize the mental health challenges faced by healthcare workers, suggesting tailored interventions to support them during crises;
- Focusing on behavioural interventions: understanding and modifying behaviours through tailored public health strategies is key to effective prevention efforts. Articles 1, 2, 7, 9 and 10 examine various behaviours (social distancing, social media use) and their mental health impacts, highlighting the need for precise monitoring and intervention strategies;
- Utilizing advanced analytical methods and implementing real-time monitoring: the use of machine learning, logistic regression, and other advanced methods allows for more accurate and timely data analysis, which is crucial for precise health surveillance. Real-time data from social media, emergency departments, and other sources enable rapid response to emerging health threats. Articles 2, 3 and 8 utilize real-time data and machine learning techniques to enhance the accuracy and timeliness of health surveillance.

Regarding innovative methods and analysis, we identified 4 groups of articles:

- Machine learning and AI: articles 2, 3 and 8 use advanced machine learning techniques, including transformer-based models and ensemble modelling, to analyse complex datasets and derive actionable insights;
- Autoregressive models: used in article 2 to analyze the temporal relationship between social media content and suicide rates;
- Topic modelling: employed in article 8 to categorize and understand discussions on social media about new substances like delta-8 THC;
- Logistic and Cox regression: utilized in several articles (1, 4, 7, 9) to analyse the impact of various factors on health outcomes.

Finally, two papers were selected to be best papers after discussions at a consensus meeting at the May 3 ^rd^ , 2024, IMIA Yearbook editorial meeting. They are listed in Table 2 . See Appendix to read the description of the two studies, their main results and their level of relevance. These two articles obtained the maximum score from a single grid of criteria based on the innovative level of the study, its methodological quality, and its scientific scope in the field of “Consumer Health Informatics”.

**Table 2.** Best paper selection of articles for the IMIA Yearbook of Medical Informatics 2024 in the section ‚Consumer Health Informatics and Education‘. The articles are listed in alphabetical order of the first author's surname. ist of the top ten keywords of the 10 selected articles.

• Niederkrotenthaler T, Tran US, Baginski H, Sinyor M, Strauss MJ, Sumner SA, Voracek M, Till B, Murphy S, Gonzalez F, Gould M, Garcia D, Draper J, Metzler H. Association of 7 million+ tweets featuring suicide-related content with daily calls to the Suicide Prevention Lifeline and with suicides, United States, 2016-2018. Aust N Z J Psychiatry. 2023 Jul;57(7):994-1003. doi: 10.1177/00048674221126649. Epub 2022 Oct 14. PMID: 36239594; PMCID: PMC10947496. • Smith BP, Hoots B, DePadilla L, Roehler DR, Holland KM, Bowen DA, Sumner SA. Using Transformer-Based Topic Modeling to Examine Discussions of Delta-8 Tetrahydrocannabinol: Content Analysis. J Med Internet Res. 2023 Dec 21;25:e49469. doi: 10.2196/49469. PMID: 38127427; PMCID: PMC10767625.

## 4. Conclusions

The integration of artificial intelligence (AI) and advanced data analytics into health surveillance marks a pivotal shift towards precision in prevention and health monitoring. By harnessing vast amounts of data from various sources, particularly social media, AI can significantly enhance the timeliness and accuracy of identifying emerging health concerns. This proactive approach is crucial in a world where rapid response to health threats can save lives and prevent widespread outbreaks. Addressing the ethical and privacy challenges associated with this approach is crucial to ensure responsible and effective implementation. As AI technologies continue to advance, their application in health surveillance will play a vital role in safeguarding public health and responding to emerging health threats promptly. Embracing this innovative approach can create a more responsive and resilient public health infrastructure better equipped to protect and promote health in the digital age.

## Notes

(2023/1/1:2023/12/31[DP] NOT “Epub ahead of print”) NOT Bibliography[pt] NOT Comment[pt] NOT Editorial[pt] NOT Letter[pt] NOT News[pt] NOT Case Reports[pt] NOT Published Erratum[pt] NOT Historical Article[pt] NOT Legal Case[pt] NOT legislation[pt] NOT (systematic review[pt] OR “review literature as topic”[MeSH Terms] OR “review”[All Fields]) AND (“consumer health informatics”[MeSH Terms] OR “consumer health information”[MeSH Terms] OR “consumer behavior”[MeSH Terms] OR “consumer attitude”[All Fields] OR “consumer organizations”[MeSH Terms]) AND (“precision prevention”[All Fields] OR “right intervention”[All Fields] OR “tailoring interventions”[All Fields] OR “tailored interventions”[All Fields] OR “illness prevention”[All Fields] OR “injury prevention”[All Fields] OR “targeted health promotion”[All Fields]) AND (“digital health”[All Fields] OR “ehealth”[All Fields] OR “e-health”[All Fields] OR “tele-health”[All Fields] OR “mhealth”[All Fields] OR “m-health”[All Fields] OR “mobile health”[All Fields] OR “telemedicine”[All Fields] OR “electronic patient-physician communication”[All Fields] OR “electronic medical record”[All Fields] “electronic health record”[All Fields] OR “personal health record”[All Fields] OR “electronic patient record”[All Fields] OR “electronic”[All Fields] OR “information technology”[All Fields] OR “communication technology”[All Fields] OR “mobile”[All Fields] OR “cell phone”[All Fields] OR “smart phone”[All Fields] OR “online”[All Fields] OR “online systems”[All Fields] OR “internet”[All Fields] OR “web”[All Fields] OR “website”[All Fields] OR “patient portal”[All Fields] OR “tele*”[All Fields] OR “digital*”[All Fields] OR “remote*”[All Fields] OR “video*”[All Fields] OR “virtual”[All Fields] OR “virtuality”[All Fields] OR “virtualization”[All Fields] OR “virtualized”[All Fields] OR “virtualizing”[All Fields] OR “virtuals”[All Fields]) AND (“social media”[All Fields] OR “facebook”[All Fields] OR “twitter”[All Fields] OR “youtube”[All Fields] OR “instagram”[All Fields] OR “social network site”[All Fields] OR “social web”[All Fields] OR “online social network”[All Fields] OR “social environment”[All Fields] OR “social process”[All Fields] OR “social competition”[All Fields] OR “social norm”[All Fields] OR “social feedback”[All Fields] OR “social influence”[All Fields] OR “social comparison”[All Fields] OR “social network”[All Fields] OR “discussion group”[All Fields] OR “support group”[All Fields] OR “social support”[All Fields] OR “community network”[All Fields] OR “online community”[All Fields])

## Notes

(2023/1/1:2023/12/31[DP] NOT “Epub ahead of print”) NOT Bibliography[pt] NOT Comment[pt] NOT Editorial[pt] NOT Letter[pt] NOT News[pt] NOT Case Reports[pt] NOT Published Erratum[pt] NOT Historical Article[pt] NOT Legal Case[pt] NOT legislation[pt] NOT (systematic review[pt] OR “review literature as topic”[MeSH Terms] OR “review”[All Fields]) AND (“precision prevention”[All Fields] OR “right intervention”[All Fields] OR “tailoring interventions”[All Fields] OR “tailored interventions”[All Fields] OR “illness prevention”[All Fields] OR “injury prevention”[All Fields] OR “Health Risk Identification”[All Fields] OR “Targeted Health Promotion”[All Fields]) AND (“social media”[All Fields] OR “facebook”[All Fields] OR “twitter”[All Fields] OR “youtube”[All Fields] OR “instagram”[All Fields] OR “social network site”[All Fields] OR “social web”[All Fields] OR “online social network”[All Fields] OR “social environment”[All Fields] OR “social process”[All Fields] OR “social competition”[All Fields] OR “social norm”[All Fields] OR “social feedback”[All Fields] OR “social influence”[All Fields] OR “social comparison”[All Fields] OR “social network”[All Fields] OR “discussion group”[All Fields] OR “support group”[All Fields] OR “social support”[All Fields] OR “community network”[All Fields] OR “online community”[All Fields])
